# Knowledge mapping of immunotherapy in castration-resistant prostate cancer: a bibliometric and visualized study (2003–2022)

**DOI:** 10.3389/fruro.2023.1239328

**Published:** 2023-10-12

**Authors:** Xianfu Cai, Chenguang Ding, Yang Li, Jin Zheng, Wujun Xue

**Affiliations:** ^1^ Department of Renal Transplantation, The First Affiliated Hospital of Xi’an Jiaotong University, Xi’an, Shaanxi, China; ^2^ Department of Urology, Mianyang Hospital Affiliated to School of Medicine, University of Electronic Science and Technology of China Mianyang Central Hospital, Mianyang, Sichuan, China

**Keywords:** castration-resistant prostate cancer, immunotherapy, bibliometrics, VOSviewer, CiteSpace, visualization analysis

## Abstract

**Objective:**

To utilize bibliometric analysis to examine the literature about immunotherapy for castration-resistant prostate cancer published within the past two decades. Through this method, we aim to visualize and analyze the research progress in this field and identify the most recent trends and developments.

**Methods:**

This research conducted a comprehensive literature review on immunotherapy for castration-resistant prostate cancer. The time frame spanned from January 2003 to December 2022, and the data were extracted from the Web of Science Core Collection database. The application of various software tools, such as CiteSpace, Bibliometrix, and VOSviewer, facilitated the visualization and analysis of the gathered data. These technological utilities illustrated the progression of prominent focus areas within the field.

**Results:**

After excluding irrelevant studies, 373 papers were selected for this study. The findings suggested that the field of immunotherapy for castration-resistant prostate cancer was rapidly developing. The USA was considered to have a significant early entrant advantage in this area and profoundly influenced the field. Similarly, China’s National Cancer center demonstrated notable advantages as a recent participant in this research domain. Major research institutions contributing to the field include the University of California, San Francisco; the University of Washington; and the Memorial Sloan Kettering Cancer Research Center. Notably, US authors James L. Gulley, Charles G. Drake, and Lawrence Fong had the largest number of publications in this area. The main research trends for immunotherapy of castration-resistant prostate cancer are membrane antigen expression, checkpoints T-lymphocyte-associated protein 4 (CTLA4) blockade, radium-223, and vaccines, and the refinement of establishing organoid models might fuel castration-resistant prostate cancer immunotherapy research in the ongoing development.

**Conclusion:**

The key trends in immunotherapy research for castration-resistant prostate cancer are membrane antigen expression, CTLA4 blockade, radium-223, and vaccines. Exploring new immune pathways and combining different therapeutic approaches to enhance immune response will be a major trend in the field in the future.

## 1 Introduction

Prostate cancer contributes significantly to global morbidity and mortality rates among men. In 2022, the USA witnessed 268,490 new prostate cancer diagnoses, with prostate cancer overtaking lung cancer as the most prevalent cancer in men, accounting for 27% of all cases. Prostate cancer was also the cause of death for 34,500 men (11%), second only to lung cancer (1). While localized prostate cancer typically presents a more favorable prognosis, metastatic prostate carcinoma has been historically regarded as an incurable condition. However, significant strides have been made in understanding and treating metastatic castration-resistant prostate cancer (mCRPC) over the past two decades (2, 3). Nine novel medications approved in the USA since 2004 for managing mCRPC are a testament to this progress. Several of these drugs have been tested and authorized for early-stage prostate cancer, including non-metastatic castration-resistant prostate cancer (nmCRPC) and hormone-sensitive metastatic prostate cancer (HRPC) (4). There were two main types of immunotherapy. The first approach seeks to bolster the immune system’s capabilities by activating it, as with vaccines and cytokines. The anti-tumor vaccine sipuleucel-T, for instance, has been approved for patients with mCRPC who exhibit no symptoms or minimal symptoms. The second approach aims to intensify T cells’ anti-tumor effect by inhibiting immunosuppressive pathways, such as those involving immune checkpoint inhibitors (ICIs). ICIs have been used to treat advanced malignant tumors and have achieved significant therapeutic effects (5). However, ICIs were not significantly effective for most patients with advanced prostate cancer, while those with responsive prostate cancer usually showed longer remissions. This indicates a potential necessity for a more extensive exploration of the impact of ICIs on prostate cancer.

Research findings indicate that during endocrine therapy most patients develop a resistance to endocrine medications, transitioning into the castration-resistant prostate cancer (CRPC) stage and ultimately advancing to mCRPC (3). Previous treatment regimens for mCRPC focused on androgen receptor pathways or paclitaxel-based chemotherapy, with a lack of effective follow-up options after progression to first-line therapy. The overall survival (OS; 15.1–18.4 months) for second-line treatment was unsatisfactory in the available studies for novel endocrine therapeutics and paclitaxel-based chemotherapeutics. New PARP inhibitors (PARPi; olaparib, rucaparib) were 33%–54.8% effective, with a median survival extension of 2.7–7.5 months (6). Immunotherapy involves activating the host’s anti-cancer immune cells to kill tumors and has been used successfully in several malignancies, including prostate cancer. In recent years, immunotherapy has received increasing attention, with the development of sipuleucel-T, programmed cell death ligand-1 (PDL-1) tumor-associated antigen-specific monoclonal antibodies, and ICIs as individualized follow-up therapy when standard treatment has failed.

CiteSpace, Bibliometrix, and VOSviewer are the most comprehensive and widely used bibliometric tools for tracking trends and hot frontiers in a particular field (7, 8). We used bibliometrics to analyze the literature related to CRPC immunotherapy over the past 20 years and to visualize the evolution of CRPC. This review visualizes the evolution of CRPC immunotherapy to predict future research directions and provide a theoretical reference basis for CRPC immunotherapy.

## 2 Methods

### 2.1 Search method

**Figure 1:**
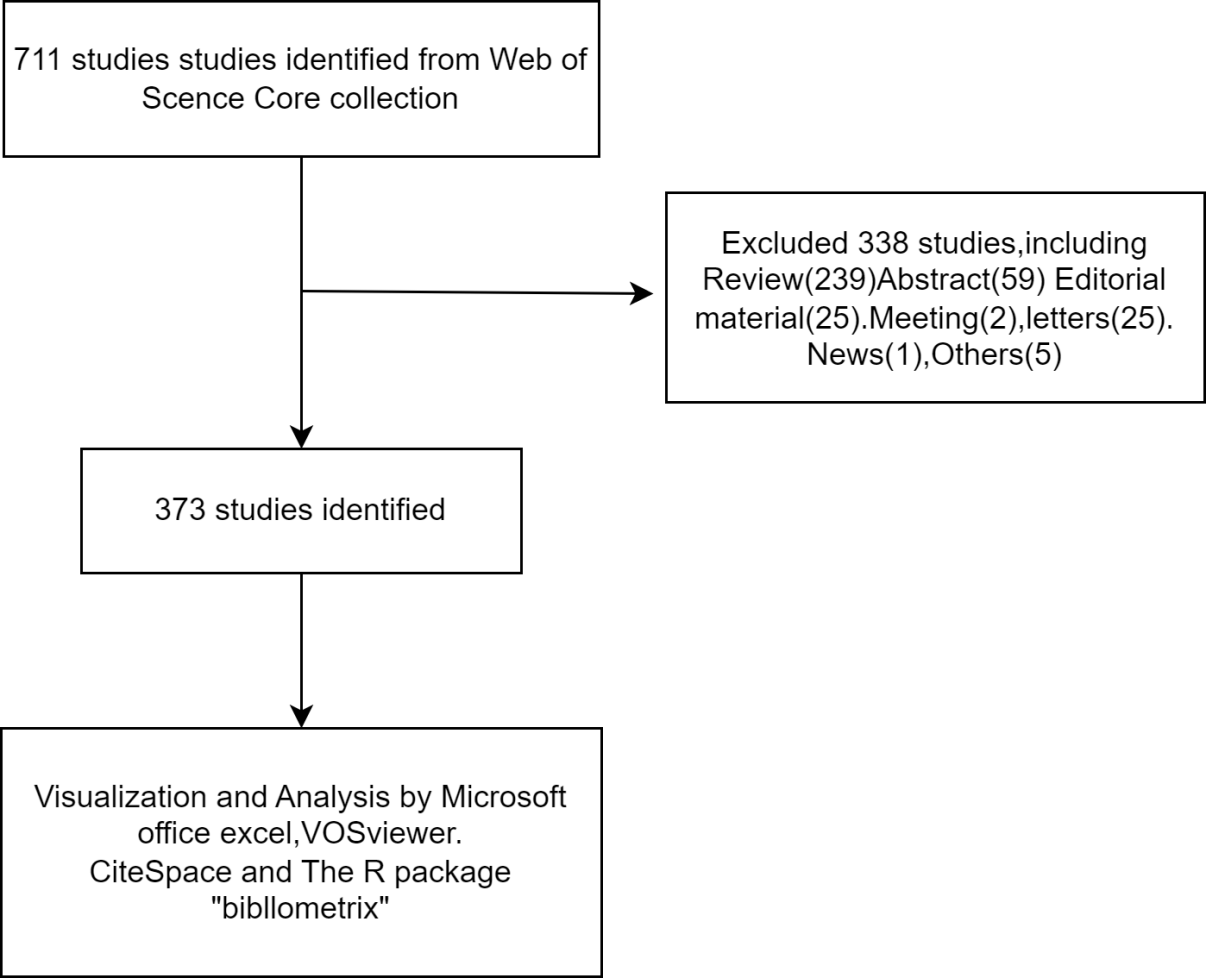
Publications screen flowchart.

The Web of Science Core Collection (WoSCC) database (https://www.webofscience.com/wos/woscc/basic-search) was used for a literature search. The search formula was [TS=(‘Castration-Resistant Prostate Cancer’) AND LA=(‘Immunotherapy’)], and the document type was set to “Article” (**Figure 1**). All literature on prostate cancer immunotherapy published from 1 January 2003 to 31 December 2022 was searched, using a combination of subject terms and free words to expand the search.

### 2.2 Data analysis

VOSviewer (version 1.6.19), a tool for analyzing bibliometric data, extracts key information from various publications (9). VOSviewer is often utilized to construct networks based on collaboration, co-citation, and co-authorship (10). In this study, the analysis performed by VOSviewer covered several aspects: country and organization analysis, analysis of journals and cited journals, analysis of authors and cited authors, and analysis of the co-occurrence of keywords. Nodes indicate elements such as countries, institutions, journals, and authors in the maps generated by VOSviewer. The size and color of the nodes indicate the number and classification of these elements. The degree of collaboration or co-citation of items is influenced by the thickness of the lines between nodes (11, 12). CiteSpace (version 6.2.R2) was another software developed by Professor Chaomei Chen for the analysis and visualization of bibliometric data (10, 13). This study used CiteSpace to create a bipartite overlay of journals. Citation bursts were used for reference analysis and keyword clustering. Country relationship maps, thematic trends, and thematic evolution were analyzed using the R package “Bibliometrix” (version 4.2.2) (https://www.bibliometrix.org) (14). Journal quartiles and impact factors were determined from the Journal Citation Report 2020. In addition, Microsoft Office Excel 2021 was utilized to analyze the publications qualitatively.

## 3 Results

### 3.1 Published volume analysis

**Figure 2:**
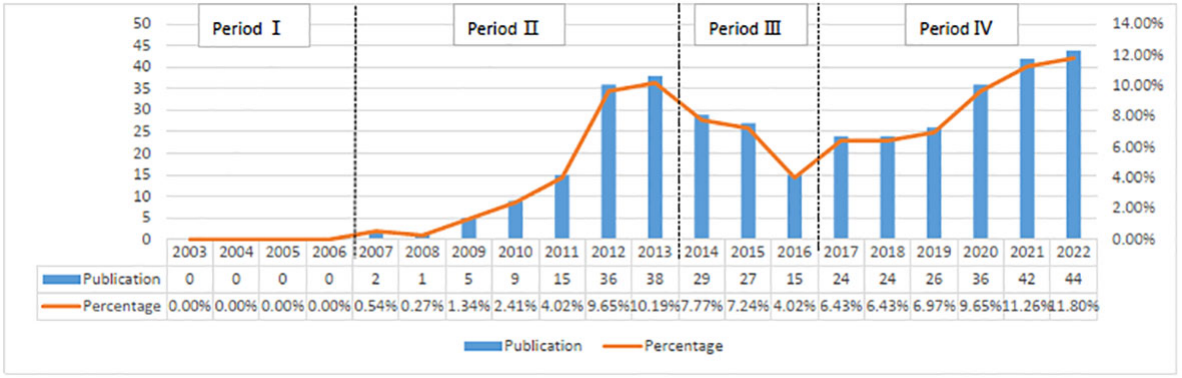
Annual immunotherapy research output for castration-resistant prostate cancer.

Based on our search methodology, there have been 711 studies on CRPC immunotherapy in the last 20 years, including 373 “articles”, 239 “reviews”, and 25 “letters”. The whole period can be divided into four parts regarding the annual increase rate in the number of publications: period I (2003–2006), period II (2007–2013), period III (2014–2016), and period IV (2017–2022). As indicated in **Figure 2**, the number of publications in the first period was 0. The number of publications in the second phase had increased yearly, averaging about 15.1 publications per year, and were of the early phases of CRPC immunotherapy research. In the third phase, immunotherapy-related studies on CRPC started to decline slightly, with an average of about 23.6 publications per year. In the fourth phase, publications increased yearly, averaging about 32.7 publications.

### 3.2 Analysis of countries and institutions

**Figure 3:**
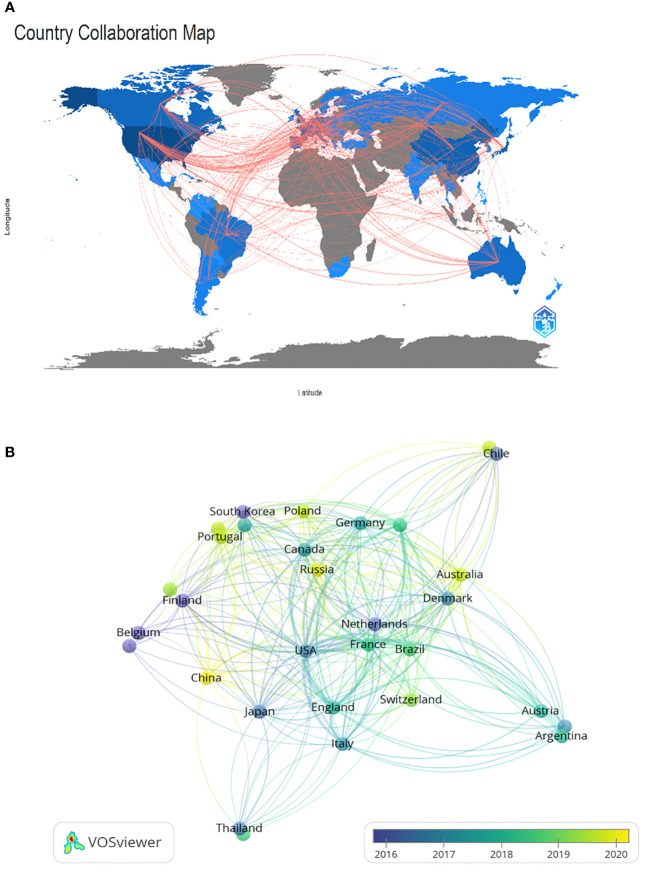
Geographical distribution **(A)** and visualization of countries **(B)** involved in researching immunotherapy for castration-resistant prostate cancer.

**Figure 4:**
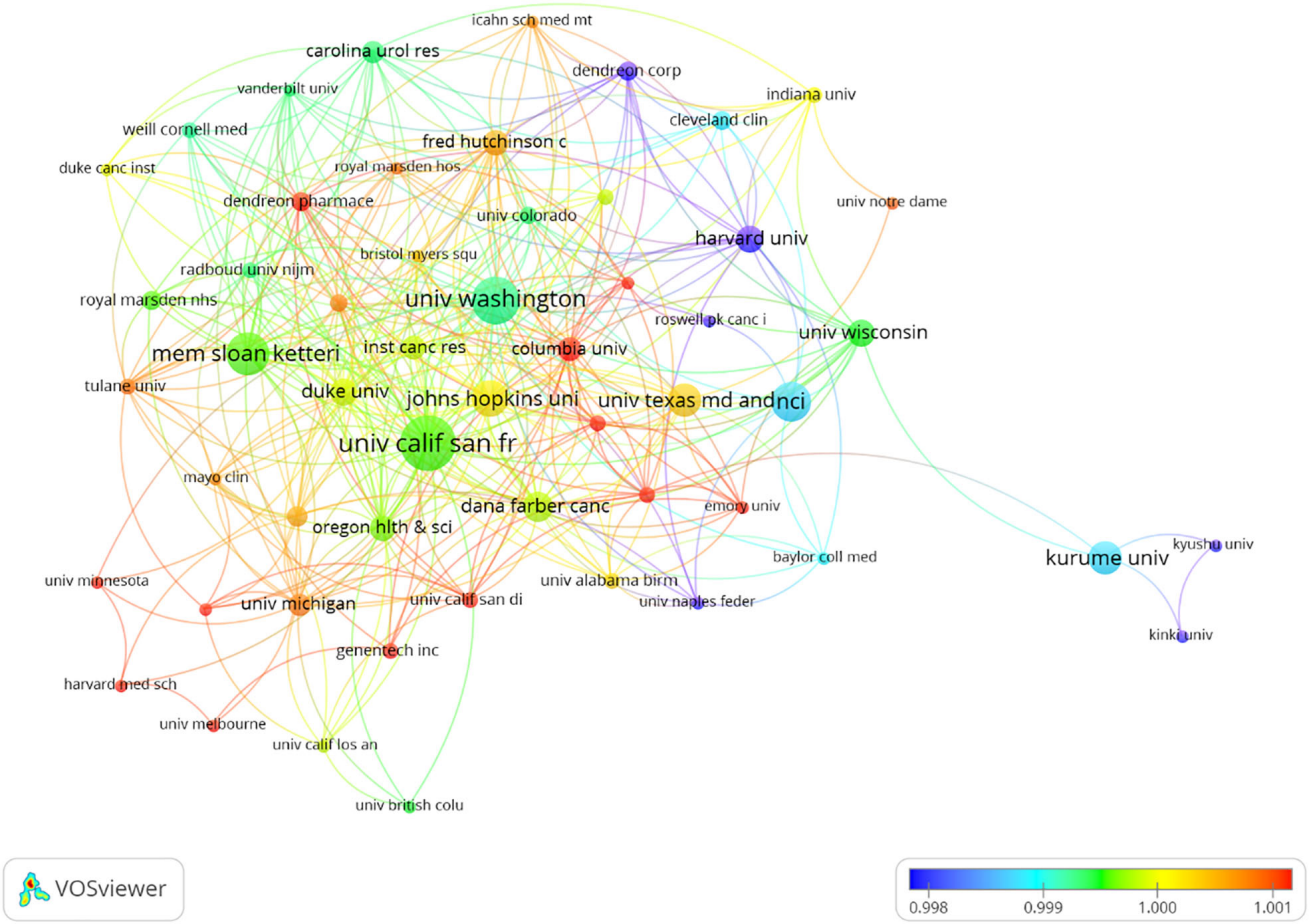
Visualization of the institutions involved in the research of immunotherapy for castration-resistant prostate cancer.

The research on CRPC immunotherapy is indeed global, with contributions from 47 countries and 788 institutions. Most publications come from Europe, North America, and Asia. Regarding the number of publications, 6 of the top 10 countries are in Europe, 2 are in North America, and 2 are in Asia (**Table 1**). The USA leads the pack, with the largest number of publications and citations (248), followed by China (34), the UK (32), and Japan (32). The comparatively small number of citations from China could be attributed to its relatively late start in this research field. Europe’s research strength and scientific output in CRPC immunotherapy are well distributed, making it a strong player in this research field. When looking at institutions, those in the USA and Japan contribute the most papers, with the USA being the most prolific. A visualized cooperation network based on the number of publications greater than two indicates active collaborations among countries (**Figure 3**). Notably, China has considerable cooperation with the USA, and the USA also collaborates extensively with Europe, Australia, and Japan. Notably, 9 out of the top 10 universities in terms of the number of papers are located in the USA, underscoring the country’s leading role in this area of research. This global cooperation and the wide distribution of research efforts encourage future progress and developments in CRPC immunotherapy. The top three organizations with the most relevant publications were the University of California, San Francisco, the University of Washington, and the Memorial Sloan Kettering Cancer Research Center. The top 10 most cited organizations were all in the USA, including Vanderbilt University, the Memorial Sloan Kettering Cancer Research Center, and Johns Hopkins University, which were very strong in this area of research (**Table 2**). We selected 54 institutions for visualization, according to a minimum of five publications, and established a collaborative network from the number of papers and the relationships between the institutions (**Figure 4**). The University of Washington, Johns Hopkins University, and the University of California, San Francisco, collaborated very closely, and Kurume University (Japan) very actively collaborated with other institutions. In addition, we noted that the University of California, San Francisco, did not have the largest number of citations despite having the most publications.

**Table 1:** Ranking of countries by number of published papers and citations.

Rank	Country	Count, *n*	Rank	Country	Citations, *n*
1	USA (America)	248	1	USA	24,449
2	China (Asia)	34	2	France	2,972
3	England (Europe)	32	3	England	2,738
4	Japan (Asia)	32	4	Denmark	1,926
5	France (Europe)	31	5	Canada	1,882
6	Italy (Europe)	29	6	Australia	1,849
7	Canada (America)	23	7	Italy	1,753
8	Germany (Europe)	20	8	Brazil	1,677
9	Spain (Europe)	19	9	Germany	1,348
10	Netherlands (Europe)	18	10	Austria	1,196

**Table 2:** Ranking of institutions by number of published papers and citations.

Rank	Institution	Count, *n*	Rank	Institution	Citations, *n*
1	University of California, San Francisco (USA)	34	1	Vanderbilt University (USA)	12,877
2	University of Washington (USA)	27	2	Memorial Sloan Kettering Cancer Center (USA)	10,660
3	Memorial Sloan Kettering Cancer Center (USA)	23	3	Johns Hopkins University (USA)	10,043
4	National Cancer Institute (USA)	21	4	University of Michigan (USA)	9,465
5	Johns Hopkins University (USA)	19	5	Dana-Farber Cancer Institute (USA)	9,416
6	University of Texas MD Anderson Cancer Center (USA)	29	6	University of California, San Francisco (USA)	5,781
7	Kurume University (Japan)	23	7	University of Washington (USA)	5,591
8	Dana-Farber Cancer Institute (USA)	15	8	Harvard University (USA)	5,246
9	Harvard University (USA)	13	9	Dendreon Corp (USA)	4,499
10	Duke University (USA)	13	10	Carolina Urologic Research Center (USA)	4,200

### 3.3 Journals and co-citations

**Figure 5:**
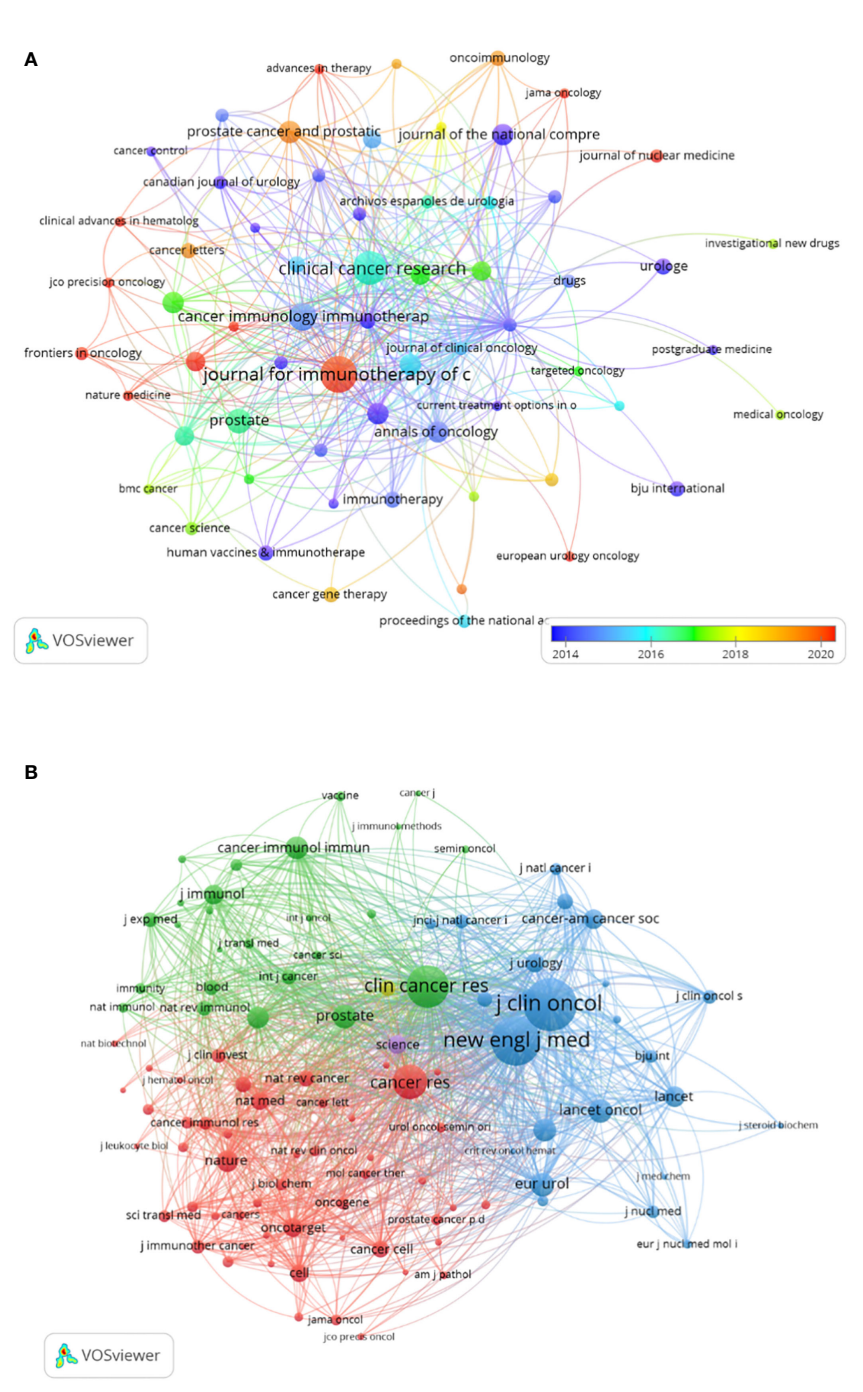
Visualization of journals **(A)** and co-cited journals **(B)** related to immunotherapy research in castration-resistant prostate cancer.

**Figure 6:**
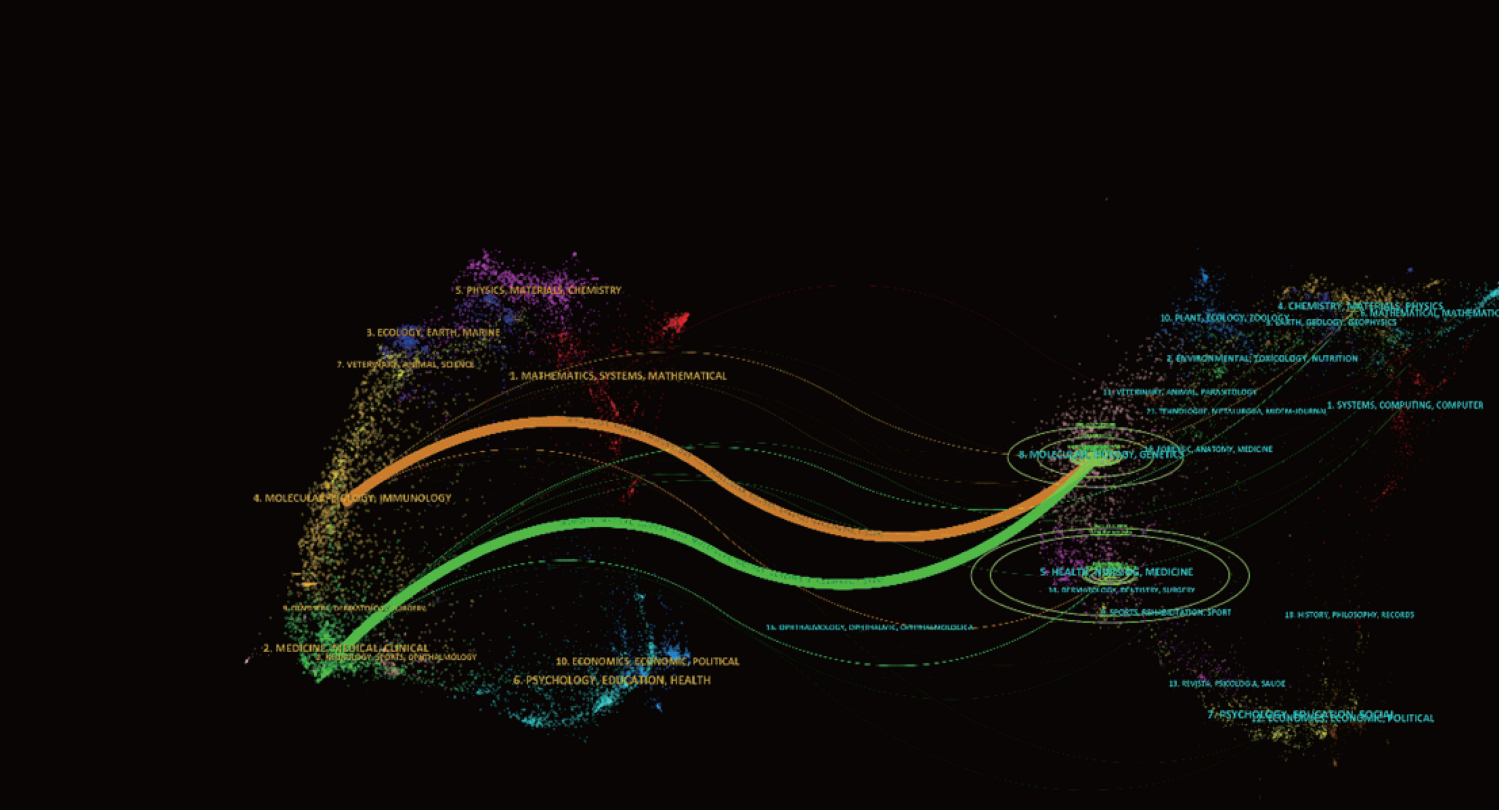
Overlapping dual-maps of journals investigating immunotherapy for CRPC.

There were 149 publications in journals related to immunotherapy in CRPC. **Table 3** shows that the *Journal for Immunology of Cancer* published the most papers (n = 25), followed by *Clinical Cancer Research* (n = 20), *Cancer Immunology Immunotherapy* (n = 14), and *The Prostate* (n = 11). The most cited of the top 10 journals was *Clinical Cancer Research* (1,428), followed by the *Journal for Immunotherapy of Cancer* (748). We then mapped the journal network by filtering 63 journals based on a minimum number of associated publications equal to two (**Figure 5A**). As shown in **Figure 5A**, the *Journal for Immunology of Cancer*, *Clinical Cancer Research*, *Journal of Clinical Oncology*, and *The Prostate* demonstrated positive relationships with their citation counts. **Table 4** shows that in terms of total citations, of the top 10 journals, 4 were cited more than 500 times, with the *Journal of Clinical Oncology* being the most cited (total citations = 1,525), followed by the *New England Journal of Medicine* (total citations = 1,310), *Clinical Cancer Research* (total citations = 965), and *Cancer Research* (total citations = 636). The highest impact factor was for the *New England Journal of Medicine* (74.699), followed by *The Lancet Oncology* (33.997). Journals with at least 20 co-citations were examined to map the co-citation network (**Figure 5B**). **Figure 5B** indicated positive co-citation associations for the *Journal of Clinical Oncology*, the *New England Journal of Medicine*, *Clinical Cancer Research*, and *Cancer*. The overlay of double graphs of journals showed the citation relationship between journals and co-cited journals, with groups of cited journals on the left and groups of co-cited journals assembled by journals on the right. From **Figure 6**, we recognized orange and green paths as the main citation paths, with studies published in molecular/biology/immunology journals primarily cited in the literature of molecular/biology/genomics journals. The most important studies published in drug/pharmaceutical clinical journals were also quoted in molecular/biology/genomics journals.

**Table 3:** Ranking of journals by number of published papers and citations.

Rank	Journal	Count, *n*	Rank	Journal	Citations, *n*
1	*Journal for Immunotherapy of Cancer*	25	1	*Clinical Cancer Research*	1,428
2	*Clinical Cancer Research*	20	2	*Journal for Immunotherapy of Cancer*	748
3	*Cancer Immunology*, *Immunotherapy*	14	3	*Annals of Oncology*	802
4	*The Prostate*	11	4	*On Target*	721
5	*Prostate Cancer and Prostatic Diseases*	9	5	*European Urology*	399
6	*Annals of Oncology*	8	6	*Cancer Immunology Immunotherapy*	668
7	*On Target*	8	7	*Prostate Cancer and Prostatic Diseases*	149
8	*Urologic Oncology: Seminars and Original Investigations*	8	8	*Future Oncology*	183
9	*Journal of the National Comprehensive Cancer Network*	8	9	*Cancer Immunology Research*	201
10	*Clinical Genitourinary Cancer*	8	10	*PLOS ONE*	227

**Table 4:** Ranking of journals by impact factor, journal citation reports category, and citation count.

Rank	Journal	Citations, *n*	Impact factor	Q
1	*Journal of Clinical Oncology*	1,525	32.956	Q1
2	*New England Journal of Medicine*	1,310	74.699	Q1
3	*Clinical Cancer Research*	965	10.107	Q1
4	*Cancer Research*	636	10.273	Q1
5	*The Lancet Oncology*	304	33.997	Q1
6	*The Prostate*	291	3.716	Q2
7	*European Urology*	283	27.424	Q1
8	*Cancer Immunology, Immunotherapy*	278	6.235	Q1
9	*Annals of Oncology*	267	18.274	Q1
10	*Proceedings of the National Academy of Sciences of the United States of America*	257	9.35	Q1

### 3.4 List of authors and cited authors

**Figure 7:**
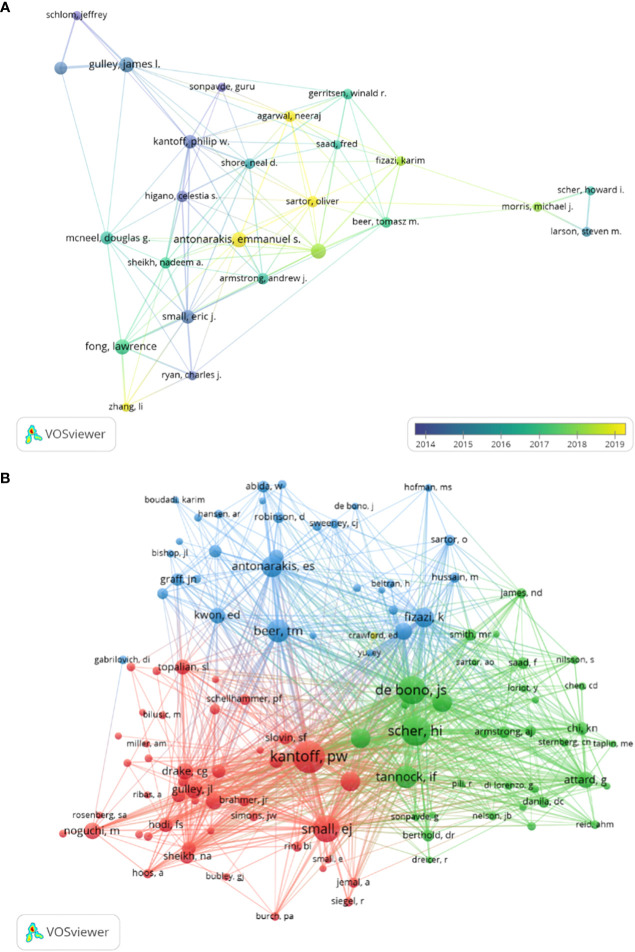
Visualization of authors **(A)** and co-cited authors **(B)** in immunotherapy research for CRPC.

CRPC immunotherapy trials involved a total of 2,363 authors. A total of 8 of the top 10 authors published more than 10 papers each (**Table 3**). Collaborative networks were constructed based on authors with seven or more publications (**Figure 7A**). Because they published the most relevant papers, James L. Gulley, Emmanuel S. Antonarakis, and others had the largest nodes. We also observe that Michael J. Morris and Tomasz M. Beer collaborated closely.

In all, 4 of the 7,333 co-cited authors had over 200 citations (**Table 5**). Philip W. Kantoff (*n* = 301) was the author with the most citations, followed by Howard I. Scher (*n* = 250) and Johonn S. de Bono (*n* = 228). To plot the co-citation network (**Figure 7B**), authors with at least 15 co-citations were filtered. There was also an important degree of collaboration between the different co-cited authors, as seen in **Figure 7B**.

**Table 5:** Top 10 castration-resistant prostate cancer immunotherapy research authors and co-cited authors.

Rank	Author	Count, *n*	Co-cited author	Citations, *n*
1	Gulley, James L.	16	Kantoff, Philip W.	301
2	Drake, Charles G.	16	Scher, Howard I.	250
3	Fong, Lawrence.	16	de Bono, Johonn S.	228
4	Antonarakis, Emmanuel S.	16	Small, Eric J.	200
5	Kantoff, Philip W.	13	Beer, Tomasz M.	151
6	Small, Eric J.	13	Tannock, Ian, F	146
7	McNeel, Douglas G.	12	Antonarakis, Emmanuel S	129
8	Madan, Ravi A.	11	Fizazy, Karim	115
9	Sartor, Oliver.	9	Petrylak, Daniel P	115
10	Hirano, Celestia S.	9	Ryan, Charles J	110

### 3.5 Reference citation burst

A reference citation burst refers to literature widely cited by scholars in a certain field over time. CiteSpace identified 12 strong citation bursts (**Figure 7B**). The bars in **Figure 7B** indicate the years, and the red bars indicate the degree of strong citation bursts (44). The reference citation burst took place from 2006 to 2022. The strongest citation burst reference (intensity = 20.09) was titled “Sipuleucel-T immunotherapy for castration-resistant prostate cancer” by Philip W. Kantoff et al. (15), with a citation burst time of 2011 to 2015.

The second most cited burst (intensity = 16.62) was “Ipilimumab versus placebo after radiotherapy in patients with mCRPC that had progressed after docetaxel chemotherapy (CA184-043): a multicentre, randomized, double-blind, phase 3 trial” by Eugene D. Kwon et al. (16) in *The Lancet Oncology*, with a citation burst from 2015 to 2019.

Recent reference citation outbreaks were published by Tomasz M. Beer et al., entitled “Enzalutamide in metastatic prostate cancer before chemotherapy” (17) and “Trial of ipilimumab versus placebo in asymptomatic or minimally symptomatic patients with metastatic chemotherapy-naïve castration-resistant prostate cancer” (18), published in *New England Journal of Medicine* and the *Journal of Clinical Oncology*, respectively.

Overall, the burst intensity of these 12 publications ranged from 8.96 to 20.09, and the endurance intensity ranged from 4 to 5 years.

In the order of the literature in **Figure 7B**, **Table 6** summarizes the main studies of the 12 publications.

**Table 6:** The 12 references with the largest number of citations by main research content.

Rank	Year	Research themes
1	2006	Sipuleucel-T potentially improved survival in HRPC patients (17)
2	2009	Sipuleucel-T was beneficial for survival in advanced prostate cancer (17, 18)
3	2010	Sipuleucel-T improves survival in mCRPC but does not affect disease progression time (19)
4	2010	PROSTVAC-VF immunotherapy showed promising evidence of reducing death rate and increasing median OS in men with mCRPC (20)
5	2010	Cabazitaxel extended survival in advanced prostate cancer (21)
6	2011	Abiraterone acetate prolonged survival in advanced prostate cancer after chemotherapy (22)
7	2012	Enzalutamide increased survival in advanced prostate cancer after chemotherapy (15)
8	2013	Abiraterone delayed cancer progression and extended survival in CRPC (16)
9	2013	Radium-223 improved CRPC OS (23)
10	2014	Ipilimumab had signs of activity with the drug that warrant further investigation (24)
11	2014	Enzalutamide delays progression and improves survival in prostate cancer (25)
12	2017	Ipilimumab has no OS improvement in advanced prostate cancer (26)

### 3.6 Hotspots and research trends

**Figure 8:**
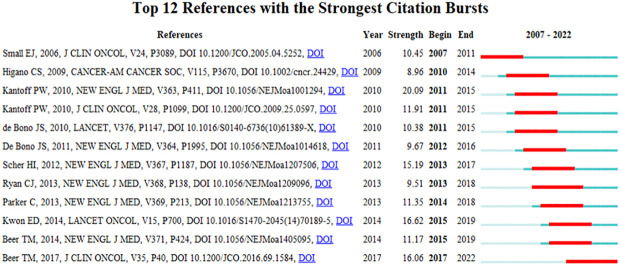
References with strong citation bursts.

Utilizing CiteSpace for keyword co-occurrence analysis and clustering facilitated the quick identification of research hotspots in the specific field. Eight clusters were obtained, representing eight key research directions, namely, prostate neoplasms, CRPC, ipilimumab, prednisone, prostate cancer, antigen, survival, and carcinoma (**Figure 8**). High-frequency keywords such as immunotherapy, prostate cancer, double-blind, survival, docetaxel, increased survival, sipuleucel-T, mitoxantrone, trial, antigen, castration-resistant prostate cancer, prednisone, enzalutamide, abiraterone acetate, ipilimumab, and abiraterone were identified, and their frequency was more than 30 times. Based on the keyword analysis of CRPC research in the past 5 years, there has been a noticeable shift in focus toward key terms such as receptors, tumors, metastasis, mechanisms, cell inhibition, tumor microenvironment, drug resistance, and checkpoints. This shows the prominent direction of research in the field of immunotherapy for CRPC.

**Figure 9:**
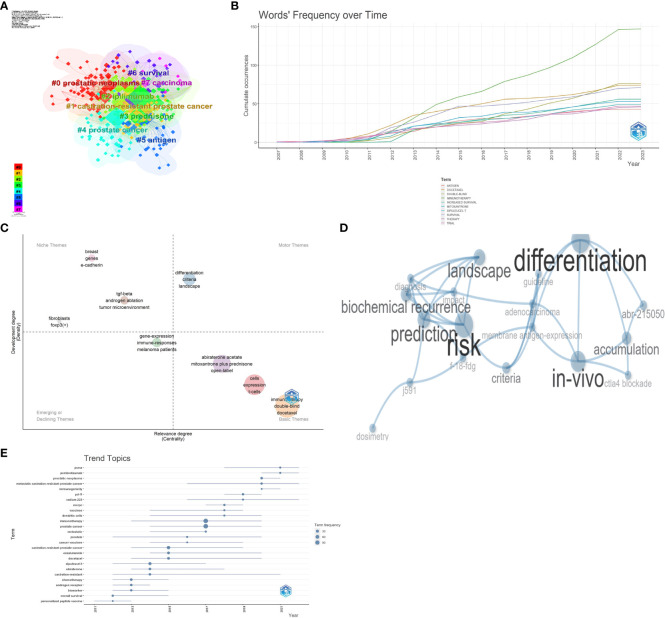
Keyword cluster analysis **(A)**, word's frequency over time **(B)**, thematic map **(C)**, motor theme cluster network **(D)**, and trend topic analysis **(E)**.

The word frequency–time relationship (**Figure 9B**) and the thematic analysis (**Figure 9C**) performed through Bibliometrix underscored that keywords such as abiraterone castration-resistant, castration-resistant prostate cancer chemotherapy, docetaxel, enzalutamide, immunotherapy, prostate cancer, prostatic neoplasms, and sipuleucel-T have been steadily increasing in frequency over time. Thematic analysis (**Figure 9C**) of relevant themes is essential for the robust development of CRPC in this research area. Further analysis (**Figure 9D**) yields hot topics such as membrane antigen expression, CTLA4 blockade, and differentiation, combined with trending themes (**Figure 9E**), PSMA pembrolizumab, radium-223, mCRPC, vaccines, dendritic cells, hot topics such as enzalutamide, docetaxel, and other hot trends. Based on these trends it is seen that currently CRPC is evolving toward immunotherapy, chemotherapy, and radiotherapy development. A comprehensive analysis could conclude that membrane antigen expression, CTLA4 blockade, radium-223, and vaccines probably represented the hot trends in CRPC immunotherapy research.

## 4 Discussion

Immunotherapy has emerged as a promising treatment option for patients with advanced prostate cancer. CRPC is a particularly challenging subtype of prostate cancer. It is often resistant to conventional therapies. Therefore, there is an urgent need to explore new research directions, such as immunotherapy, for CRPC patients. This study aims to provide a comprehensive visual analysis of progress in immunotherapy for CRPC. Our study reveals a significant progression in research related to immunotherapy for CRPC over the past two decades. The timeline of this research can be partitioned into four distinct phases, characterized by a steady increase in the annual publication rate. This trend is projected to continue in the future.

In the global landscape, the USA, China, the UK, and Japan emerged as front runners in CRPC immunotherapy research. The USA led the pack, with the most publications and citations. However, China’s citation count was low, which might be due to its later entry into CRPC immunotherapy research. Europe demonstrated well-rounded research competence and significant contributions to CRPC immunotherapy, underscoring its stature as a potent research hub in this domain. A notable number of US research institutions exhibited substantial research capabilities in this area.

The principal authors in this field clustered into four groups with robust internal connections, suggesting the formation of stable collaborative networks within CRPC immunotherapy research. The five most cited articles, demonstrating a citation burst, reflected the high-quality clinical research on immunotherapeutic agents for CRPC.

Investigations into journals and co-cited journal analysis revealed a primary focus on the molecular, biological, and genomic aspects of CRPC immunotherapy research. Most of these studies pertained to therapeutic vaccines, particularly sipuleucel-T and PROSTVAC-VF. Sipuleucel-T (Provenge^®^, Dendreon, USA), despite being a U.S. Food and Drug Administration-approved immunotherapy product, had limited benefits, a high cost, and was controversial. Clinical trials have indicated that sipuleucel-T treatment did not significantly prolong the time of progression but safely prolonged OS by a mean of 4 months (3). PROSTVAC-VF, despite the results of Phase II studies showing a reduction in mortality in mCRPC patients, was not shown to have an impact on median OS in mCRPC in Phase III clinical trials (27). Over the past two decades, therapeutic cancer vaccines that target single or multiple antigens using various methods have been extensively studied to treat prostate carcinoma. However, most of these were confined to Phase I trials. Although most vaccines were safe, with indications of immunological activity, exclusive use of vaccines targeting the amplification and/or activation of tumor-responsive T cells did not improve OS in patients, indicating their insufficiency for treating advanced prostate cancer, according to Phase III trials evaluating different vaccine strategies (GVAX, tumor lysate-loaded dendritic cells, peptide vaccines, viral vaccines) (28). The above studies illustrated that nucleic acid vaccines had a low cost, were easy to manufacture, had safety advantages, and were less restricted to certain major histocompatibility complex types than peptide vaccines. The success of the SARS-CoV-2 vaccine demonstrated the great potential of RNA and DNA vaccines in terms of safety and stability. Nevertheless, many vaccines did not exhibit sufficient clinical activity to proceed to randomized clinical trials.

Combining PARPi and immunotherapy offered clinicians novel treatment perspectives. PARPi application can reshape the tumor immune microenvironment by increasing the tumor mutation burden and activating the stimulator of interferon genes (STING) pathway, thereby enhancing immune activation. The synergistic effect between increased tumor mutation burden and PDL-1 expression in ICI therapy attracted more effector T cells to infiltrate the tumor immune microenvironment, inhibiting tumor growth and metastasis (29). Research on preclinical models has also revealed that inhibiting DNA damage repair through PARPi could increase new antigen load and mutation burden, thereby improving the response to PDL-1 immunotherapy. Unrepaired DNA damage led to tumor-derived DNA damage, and cytoplasmic DNA sensors detected double-stranded DNA, activating the STING signaling pathway. STING activation stimulated the production of antiviral interferon type I interferon, promoting systemic immune responses and regulating antitumor immunity involving T cells, natural killer cells, and dendritic cells (30). PARPi could upregulate PDL-1 expression by inactivating Glycogen synthase kinase-3 beta, increasing tumor cell toxicity while inhibiting T-cell activation. These findings formed the basis for recently published clinical trials evaluating the combination of avelumab (an anti-PDL-1 antibody) and talazoparib (an oral PARPi). These trials aimed to identify patient populations that might benefit from this combination therapy, with initial results suggesting significant responses in patients with breast cancer gene 1/2 mutation tumors. These studies were of great significance for understanding the mechanism and patient population that might benefit from the combination therapy of PARPi and ICIs (31). Our previous clinical studies, analyzed through metric software, echoed these findings, focusing primarily on sipuleucel-T and PROSTVAC-VF in CRPC immunotherapy research. Sena et al. proposed that the characteristics of prostate cancer and its host environment might make it universally resistant to CTLA4 and PDL-1 blockers (32). A single-arm pilot study showed that a CTLA4 blocker (tremelimumab) combined with a PDL-1 blocker (durvalumab) was safe and well tolerated in mCRPC patients who had not received chemotherapy (33). Radiotherapy and chemotherapy would release new antigens by killing tumor cells, leading to cross-priming of additional antigen-specific T cells (34). These findings are congruent with keyword and thematic hotspot analysis trends in CRPC immunotherapy research, such as membrane antigen expression, CTLA4 blockade, radium-223, and vaccines, among other related research directions.

Despite extensive research on CRPC immunotherapy, a disparity persists between clinical and basic research, with a shortage of studies focusing on validating animal models possibly accounting for this gap. Notably, creating animal models of CRPC presents certain challenges. When comparing cell-derived xenograft models, patient-derived tumor xenograft (PDX) models exhibit superior histopathological attributes. Retaining most primary tumor characteristics at histopathological, molecular biological, and genetic levels, the PDX model showcases enhanced predictive capability for clinical efficacy. However, it is not entirely clear to what extent PDX and prostate cancer organoids can emulate essential features of therapeutic resistance and drug response (35). Owing to severe immunodeficiency models, the metastatic behavior of cancer cells in PDX models differs from the clinical scenario, imposing limitations on the application of these models. These models also carry drawbacks, such as prolonged preparation time, high costs, and an inability to perform high-throughput drug screening (36). Patient-derived tumor organoids (PDOs), a significant aspect of organoid technology, can effectively simulate intra- and inter-tumor heterogeneity and hold considerable potential for CRPC immunotherapy research. However, as PDOs are only tissues and lack vascular and neural structures, they fall short of completely mimicking the physiological functions of human organs (37). Hence, developing models that can fully emulate the physiological functions of human organs remains a crucial task for future research in prostate cancer immunotherapy.

Naturally, this study had some shortcomings. First, the data in this study came only from the WoSCC database, ignoring other databases, which might have meant that some relevant studies were missed and might have caused selection bias. Second, we focused on studies published in English only, which could lead to an underestimation of non-English publications. Finally, we excluded publications from 2023 onwards because of a lack of data.

## 5 Conclusion

This article provided an analysis of publications in the field of immunotherapy in CRPC, which can help researchers understand the trends and international research progress in this field. The increasing number of publications indicated that the study of immunotherapy in CRPC is attracting more and more attention from researchers globally. Prominent contributors to this research domain are from the USA and European nations, and China, although a late entrant, has shown rapid development. Nevertheless, the communication and collaboration between different countries and organizations need to be strengthened. Investigations focusing on the intrinsic mechanisms of the role of immunotherapy in CRPC development, such as immune checkpoint detection, membrane antigens, and biomarkers, could play a critical role in vaccine development, and researchers and policymakers new to the field can access a comprehensive overview of its evolution and the latest advancements.

## Data availability statement

The original contributions presented in the study are included in the article/supplementary material. Further inquiries can be directed to the corresponding authors.

## Author contributions

CX was responsible for the research design and data collection and the analysis and interpretation of the experimental results. He also conducted a literature review and wrote the paper. DC participated in the research design and data collection, conducted statistical analysis, designed and produced tables and figures, and revised the paper. LY developed and implemented the research plan, provided result interpretation, participated in the literature review, and contributed to writing the paper. ZJ contributed to the experimental design and data collection, conducted statistical analysis, designed and produced tables and figures, and revised the paper. XW coordinated the entire research process, including research design, project execution, data analysis and interpretation, and paper writing. Overall, CX, DC, LY, ZJ, and XW worked together to complete the research, with each author taking on different tasks and responsibilities and making important contributions to the final research results.
